# Bromoform Poisoning

**Published:** 1910-06

**Authors:** Rupert Waterhouse

**Affiliations:** Assistant Physician, Royal United Hospital, Bath.


### BROMOFORM POISONING.

BY

RUPERT WATERHOUSE, M.D., M.R.C.P. Lond.,
*Assistant Physician, Royal United Hospital, Bath.*

---

BROMOFORM, first produced by Nunneley and Suchard about the year 1840, by the action of bromine on alcohol in the presence of an alkali, was, some twenty years later, tried by Rabuteau and others as a general anæsthetic, but does not appear to have been used much for this or for any medicinal purpose until, in 1889, S. K. Stepp,[1] of Nuremberg, advocated it as a remedy for whooping cough.

He related a series of sixty-five, which in a second communication he brought up to a hundred, cases treated by this drug with most satisfactory results, claiming that it had a very decided influence in cutting short the disease and in preventing complications, the longest time taken for complete cure being four weeks, and that, in no instance, did any disagreeable effects follow its use. He credited the drug, also, with considerable prophylactic power.

This opinion as to its utility in whooping cough was speedily confirmed by Neumann, Fischer, Löwenthal, Cassel, Burton-Fanning and many other investigators.

Fischer,[2] after using it in fifty-one cases, came to the conclusion that " there is no question that it is the best known remedy when properly applied ; " whilst Löwenthal,[3] who treated by this means a hundred children in Professor Senator's clinic in Berlin, regarded its action as " almost specific," at all events if used from the commencement. But both he and Burton-Fanning, who, from an experience of thirty cases, considered the remedy a very valuable one, drew attention to the occasional occurrence of toxic effects.

Bromoform, whose chemical formula is represented by $CHBr_3$, has the same composition as chloroform and iodoform, with the chlorine and iodine radicles, respectively, replaced by bromine. It is a colourless, mobile liquid with a sweet taste and an odour which, though recalling those both of chloroform and iodoform, is rather pleasant than otherwise. The specific gravity is 2.83 ; it is freely soluble in alcohol, ether and almond oil, less soluble in glycerine (1 in 80) and only slightly in water (1 in 800). When exposed to sunlight it decomposes and becomes yellow from liberation of bromine.

Its great weight and its slight solubility in water render it a difficult drug to dispense, as in any mixture containing water, or even mucilage, bromoform, being heavier than either, will sink to the bottom. Even when six minims are dissolved in six drachms of rectified spirit and water is gradually added up to six ounces, the mixture being well shaken after each addition, a considerable quantity of the drug will be found, after standing, as a deposit at the bottom of the bottle.

The doses which have been prescribed have varied considerably. The German pharmacopœia of 1900 gives .5 grammes (about 3 minims) as the maximum single and 1.5 grammes as the maximum daily dose.

Stepp recommended five to twenty drops in twenty-four hours in very frequent doses, and, in his second communication, one drop three times a day to children from three to four weeks old, four or five drops three or four times a day to children from two to three years, and six to seven drops three or four times a day to children above seven.

It must be borne in mind, in connection with these doses, that there are about eight " drops " of bromoform, according to the measurements decided upon at the International Congress for the Unification of Pharmacopæial Formulæ of Patent Drugs and Preparations, held at Brussels in 1902, to a minim.

Löwenthal gave from two to five drops three times a day. Fiertz[4] for children under ten, recommended as many drops as the child had years of age, plus two, every six hours; and Marpan,[5] in children under five, gave four drops daily for each year of age, and in older children twenty drops daily, and advised gradually increasing the doses till they were doubled.

Burton-Fanning advised half a minim three times a day for children under one year, one minim for children between one and three, and two minims for those between three and six, these doses being gradually increased till doubled.

More recently Voelcker[6] says that, in whooping cough, "bromoform in doses of five minims is sometimes useful," whilst Parkinson[7] finds that the paroxysmal attacks are greatly lessened in severity and vomiting, if previously present, is entirely prevented by doses of from one-eighth to three quarters of a minim.

Instances of poisoning by this drug fall into three groups.

In the first are those in which, either wilfully or by misadventure, a very large amount has been taken at one time; in the second, those in which toxic symptoms have followed the administration of the *last dose* in a bottle of mixture containing it; and in the third, those in which apparently only the quantity that has been prescribed has been taken.

Those in the first group are mostly children who, liking the sweet flavour of the drug, have gained access to the medicine bottle and freely indulged their taste.

Thus Dwelle records the death of a child two years old who, whilst taking two drops of bromoform every four hours, obtained the bottle and drank about half its contents, in this way taking from thirty to forty drops of bromoform at one dose. Medical assistance could not be procured for an hour and a half, and at the end of that time the child was quite insensible, with dilated pupils and breath smelling strongly of bromoform; no pulse could be felt, and the heart sounds were not audible. Despite strychnine and whisky hypodermically, lavage, and artificial respiration, the child died about three hours later.

Tresling reports the death of a child, aged four years, seven and a half hours after a drachm of bromoform was given to it by mistake, and other fatal cases are recorded by Nauwelaers, Leurns and Müller.

Darling records a case of recovery in a girl of six after taking one and a half drachms of pure bromoform. The stomach was washed out for an hour and a half until the washings were quite free from the odour of bromoform, and sal volatile and strong coffee were administered by rectum and through the stomach tube.

Similar examples are related by Sachs, Pannwitz, Nolden (two), Schliepers, Börger (two), Bömmel, Czygan and others.

Cases in the second group, namely those in which symptoms of poisoning occur after taking the *last dose* of a mixture containing bromoform, are described by Dean, Stone, Evans, Burton-Fanning (four cases), Stokes (two cases), Benson and Langdon. In all of these recovery took place; but in 1898 an inquest was held at Lancaster on a girl, aged five years, who died after swallowing the last dose of a mixture which had contained thirty-six minims of bromoform in mucilage and water. The inquest is referred to in the *Lancet* of 1898, but further details were not forthcoming.

In the case described with much humour by Stone, a child of two was left for dead all night (according to another practitioner who was summoned, of "black diphtheria"), but eventually recovered so rapidly that in a few days "she whooped as loudly as ever."

The writer began to use bromoform in the treatment of whooping cough in 1902 whilst resident medical officer at the Royal United Hospital. The results were found to be decidedly superior to those attained by the use of the remedies in common use, especially in diminishing the number and violence of the paroxysms and in checking vomiting. A noticeable feature, too, in this and in later experience has been the rarity of bronchial and pulmonary complications, though as the frequency and severity of these vary so greatly in different epidemics, this impression, received from data so unreliable as those of out-patient practice, may be erroneous.

In January, 1903, bromoform was prescribed, amongst others, for three children of German parentage. The drug was dispensed in a mixture which went by the name of mistura bromoformi, prepared by adding bromoform dissolved in spirit and glycerine to mucilage and water. It fell to the lot of the eldest child, a boy four and a half years old, taking rather less than one minim three times a day, to have the last dose out of a bottle common to the three. Almost immediately he became faint and ill, vomited and turned very drowsy. When seen neary an hour later he lay completely unconscious, breathing stertorously with eyes closed, contracted pupils and rapid, feeble pulse. His breath and the vomited material both smelt strongly of bromoform. The stomach was thoroughly washed out until the washings were free from the smell of bromoform, stimulants were administered, and in about four hours the boy had recovered consciousness and was able to return home. Although the importance of thoroughly shaking the bottle before each dose was emphasised in this, as it was in every case, it was thought probable at the time that, in consequence of the mother's imperfect acquaintance with the English language, she had not comprehended the directions given, though this she strenuously denied, declaring that she had never omitted to do so.

However, in view of the possibility of another such accident, the use of bromoform in the out-patient department was discontinued until during a rather severe epidemic of pertussis in 1906, as no remedy seemed so efficacious in controlling the disease as bromoform had been, and it being learnt on consultation with our hospital dispenser, Mr. Snow, that a very satisfactory emulsion could be obtained by the use of tincture of senega, as recommended in Martindale and Westcott's *Extra Pharmacopœia,* the use of the drug was resumed, and it was employed in a large number of cases without any unpleasant effects arising until the following case occurred.

A little girl, five years old, attended hospital on March 17th, 1910, suffering from moderately severe, uncomplicated whooping-cough. A teaspoonful (containing nearly one minim of pure bromoform) of a mixture made according to the following formula—

| | | |
|---|---|---|
| Bromoform .. | .. | ʒ xi ♏ 44 |
| Tr. Senegæ .. | .. | ʒ iii ss. |
| Spt. vin. rect. | .. | ʒ i |
| Syr. Aurantii | .. | ʒ iv |
| Aq. ad .. | .. | ʒ xliv |

was prescribed, to be taken three times a day. On March 24th the child was, according to the mother, better, but for three or four days had complained of pain in the front of the neck. Nothing abnormal was to be seen inside the throat, but a very distinct soft uniform enlargement of the thyroid gland was discovered, slightly tender on manipulation. Red iodide of mercury ointment was given to be rubbed into the swelling once a day, while the bromoform mixture was repeated. On March 31st the thyroid swelling was less, and the child was progressing satisfactorily. A fresh bottle of the mixture was prescribed, and a dose was given about 2 p.m. The child then went, as was her custom, to lie down. About 6 p.m. her mother found her sleeping very heavily, and when with difficulty she was aroused she vomited and staggered about. After this she again became very drowsy, and Dr. Heathcote was summoned. He found the child quite insensible, and diagnosing the condition, from the history and symptoms, as one of bromoform poisoning, immediately dispatched the patient to hospital, and very kindly telephoned to me, so that I was able to see the child very shortly after her arrival there. She was then deeply unconscious, colour fair, lips bluish, pupils contracted but not pin-point. There was very marked flaccidity of the skeletal muscles; the corneal, patellar and plantar reflexes could not be obtained. There was a faint sweetish ethereal odour to the breath; pulse 120, temperature 96° F. The condition bore a striking resemblance to that of a child in a state of chloroform anæsthesia.

Although it was some six hours, according to the mother's statement, since the dose had been taken, it was thought advisable to wash out the stomach. As the tube passed the larynx it excited coughing and two or three distinct "whoops." The stomach was empty and the washing clear and odourless. Strychnine was administered hypodermically and the rectum washed out, and consciousness was soon regained, and on the following day the child seemed none the worse for its alarming experience.

The explanation of the poisonous effect of the drug in this case is not clear, and unfortunately the remainder of the bottle could not be obtained for analysis. There seem, however, to be three possibilities: first, that there was an idiosyncrasy to the drug; secondly, that a quantity greater than that supposed was taken; and thirdly, that the medicine had decomposed.

The first seems to be negatived by the fact that the girl had already taken the medicine for a fortnight without showing any special susceptibility thereto, unless, indeed, the enlargement of the thyroid is to be regarded as the expression of a reaction against the drug rather than against any other poison.

The second appears unlikely. Several other children were supplied from the same stock bottle without any untoward effects accruing, whilst the mother was sure that the child only had a teaspoonful of the mixture, and that she had not helped herself later, especially as she had a decided dislike for the mixture.

With regard to the third possibility, the mixture is already yellow, from the presence of tincture of senega, and the slight alteration of colour produced by the liberation of bromine would probably not be noticeable, but the symptoms were those typical of bromoform rather than those of bromine poisoning.

Very similar symptoms were, however, observed in a case mentioned by Oberdorfer of a child, four and a half years old, who took five drops of bromoform three times a day for a week. The order was then repeated for the next week, but on the second day, just after a dose, the child became comatose, with rapid, feeble pulse and stertorous breathing. The fluid in the bottle was found to have separated into two layers, a lower "muddy," and an upper oily and viscid. Analysis showed that it consisted of bromine and hydrobromic acid. Burton-Fanning also mentions two patients in whom drowsiness and slight intoxication were ascribed to bromoform which had been allowed to decompose.

Other instances in which symptoms of severe poisoning occurred after relatively small doses are mentioned by Dillard in a child, aged sixteen months, to whom a dose of four drops was given and repeated in two hours' time, and by Lobl in a child, aged seven months, to whom only five drops were given, and in a boy, aged two years, who took four drops, followed, through an error, in an hour's time by another four.

On the other hand, Charpentier mentions the case of a child, of two years, who took as much as thirty-four drops a day without any ill-effects.

In nearly all the recorded instances of bromoform poisoning symptoms have been observed to commence almost immediately after the peccant dose has been taken.

Cheney, however, records the case of a girl, three years old, taking two drops of bromoform every four hours, who having lunched heartily at 1 p.m. took the last dose of her medicine, which was observed to look oily, at 2.30. All the afternoon she seemed perfectly well, being out of doors walking, but at 5.30 she complained of giddiness, fell down and passed into a condition resembling intoxication, followed by complete unconsciousness, from which she finally awoke, after appropriate treatment, at 8.30.

When symptoms of bromoform poisoning have arisen they have consisted, in mild cases, of headache, faintness, giddiness, uncertain gait, incoherent speech, causeless laughter and drowsiness.

In severe cases there has been insensibility, pallor, cyanosis, coldness of the surface, loss of the corneal and other reflexes, anæsthesia, weak, irregular, rapid pulse, retching, vomiting and, in Benson's case, involuntary defæcation. The breathing may be stertorous or shallow or may fail entirely, so that artificial respiration is imperative. The pupils are generally stated to be contracted or pin-point, but in a few cases were dilated, and in one of Burton-Fanning's were unequal. The skeletal muscles are, as a rule, described as flaccid or relaxed, but some authors lay stress upon an exception in the case of the masseters, which are in several instances mentioned as being contracted. Spasms and cramps have also been met with in the limbs.

As a rule, the characteristic bromoform odour pervades the breath, and may, according to Hasemann, be detected as late as twenty-four hours after the drug has been swallowed, whilst in Evans's case there was a sweet smell to the breath for several days.

The treatment adopted has included the use of emetics, lavage of the stomach and bowel, counter-irritation over the kidneys, friction and slapping of the skin, elevation of the feet, application of cold water to the chest, hot fomentations to the heart, the introduction of strong coffee, brandy and ammonia into the stomach and rectum, strychnine, ether, whisky and atropine hypodermically, strong ammonia to the nostrils, oxygen, faradism, rhythmic traction on the tongue and artificial respiration.

*Post-mortem* examinations have disclosed nothing characteristic. Hyperæmia of the brain and of the gastric and rectal mucosa has been met with, and in Müller's case the blood is described as dark red and fluid; in this case 1.16 grammes of bromoform were recovered from the gastro-intestinal tract after death.

It will be seen from the foregoing that whilst those who have had considerable experience of this drug are almost unanimous in giving a favourable verdict on its influence in controlling whooping cough, the remedy is not without considerable potentiality for evil.

In the first place is the risk common to all poisonous drugs—for instance, belladonna or butylchloral hydrate—of a large overdose, a risk rather greater, perhaps, in the case of bromoform on account of the partiality some children display for it.

Secondly is the danger, when the drug is prescribed in mixture, of the *last dose,* a danger diminished but not entirely obviated, as is shown by the case recorded by Benson, by emulsifying the drug with tincture of senega.

So alive was Burton-Fanning to this peril, that in addition to impressing upon his out-patients the importance of thoroughly shaking the bottle, he counselled them always to throw away the last dose.

A still better plan, doubtless, would be to follow what is, to judge from the literature, the almost universal practice now on the continent, and let the required number of drops of pure bromoform be dropped each time into a teaspoonful of water, or to give the drug in capsules.

| | Sex. | Age. | Quantity of Bromoform taken. | Result. | Recorded by | Reference. |
|---|---|---|---|---|---|---|
| 1 | ♀ | $1\frac{1}{4}$ | 2 grammes .. .. .. .. | .. | Löwenthal .. .. | *Berl. klin. Wchnschr.*, 1890, xxvii, 508. |
| 2 | | 4 | $1\frac{1}{2}$ grammes .. .. .. | .. | Sachs.. .. .. .. | *Therap. Monatsh.*, 1890. |
| 3 | ♂ | $4\frac{1}{2}$ | 20 drops .. .. .. .. | .. | Pannwitz .. .. | *Ibid.* |
| 4 | | $1\frac{3}{12}$ | .... | D | Nauwelaers .. .. | *Rev. mens. d. Mal. de l'Enf.*, 1891, Feb. |
| 5 | ♀ | 3 | 6 grammes .. .. .. .. | .. | Nolden .. .. .. | *Therap. Monatsh.*, 1892. |
| 6 | ♂ | $2\frac{1}{2}$ | 4 grammes .. .. .. .. | .. | Nolden .. .. .. | *Ibid.* |
| 7 | | $\frac{3}{12}$ | Last dose .. .. .. .. | .. | Platt .. .. .. | *Times and Reg.* (Phila.), 1892, xxiv, 651. |
| 8 | | .. | ? .. .. .. .. | .. | Burton-Fanning .. | *Practitioner*, 1893, l, 100. |
| 9 | | .. | Drug decomposed .. .. | .. | Burton-Fanning .. | *Ibid.* |
| 10 | | .. | Drug decomposed .. .. | .. | Burton-Fanning .. | *Ibid.* |
| 11 | ♀ | 4 | Last dose .. .. .. .. | .. | Dean .. .. .. | *Lancet*, 1893, i, 1062. |
| 12 | .. | .. | .... | D | Leurns .. .. .. | Die Hebenwirkungen der Arzneimittel, 1893. |
| 13 | ♂ | $5\frac{1}{2}$ | 20 drops .. .. .. .. | .. | Schliepers .. .. | *Therap. Monatsh.*, 1894. |
| 14 | ♀ | 2 | 10 grammes between the | .. | Fiertz .. .. .. | Inaug. Dissert., Zurich, 1894. |
| 15 | ♀ | 4 | two in 24 hours .. .. | .. | Fiertz .. .. | *Ibid.* |
| 16 | | $\frac{10}{12}$ | 50 drops .. .. .. .. | .. | Bömmel .. .. | *Deutsche med. Wchnschr.*, 1896, xxii, 46. |
| 17 | ♂ | $2\frac{1}{2}$ | 5–7 grammes .. .. .. | .. | Czygan .. .. .. | *Ibid.* |
| 18 | ♂ | 5 | 15–20 drops .. .. .. | .. | Börger .. .. .. | *München. med. Wchnschr.*, 1896, xliii, 469. |
| 19 | ♀ | 3 | 5 grammes .. .. .. .. | .. | Börger .. .. .. | *Ibid.* |
| 20 | | $4\frac{1}{2}$ | 4–5 grammes .. .. .. | .. | Szegvári .. .. | *Gyogyaszat* (Budapest), 1897, xxxvii, 304. |
| 21 | ♀ | 3 | Last dose .. .. .. .. | .. | Cheney .. .. .. | *Arch. Pediat.*, 1897, xiv, 112. |
| 22 | ♂ | 2 | 5–6 grammes .. .. .. | D | Müller .. .. .. | *München. med. Wchnschr.*, 1898, xlv, 1211. |
| 23 | | .. | .... | .. | Reinecke .. .. | 1898. |
| 24 | ♀ | 5 | Last dose .. .. .. .. | D | ? | *Lancet*, 1898, ii, 1816. |
| 25 | | .. | .... | .. | Kirste .. .. .. | *München. med. Wchnschr.*, 1899, xlvi, 1134. |
| 26 | | $4\frac{3}{4}$ | .... | .. | Schmitt.. .. .. | *Ibid.*, p. 149. |
| 27 | ♀ | 2 | Last dose .. .. .. .. | .. | Stone .. .. .. | *Boston M. and S. J.*, 1899, cxl, 160. |
| 28 | ♀ | 8 | Last dose .. .. .. .. | .. | Evans .. .. .. | *Lancet*, 1899, i, 119. |
| 29 | | .. | Last dose .. .. .. .. | .. | Burton-Fanning .. | *Ibid.*, p. 189. |

| | Sex. | Age. | Quantity of Bromoform taken. | Result. | Recorded by | Reference. |
|---|---|---|---|---|---|---|
| 30 | | .. | Last dose .. .. .. .. | .. | Burton-Fanning .. | *Ibid.* |
| 31 | | .. | Last dose .. .. .. .. | .. | Burton-Fanning .. | *Ibid.* |
| 32 | | 4 | Last dose .. .. .. .. | .. | Stokes .. .. .. | *Brit. M. J.*, 1900, i, 1,283. |
| 33 | | 2 | Last dose .. .. .. .. | .. | Stokes .. .. .. | *Ibid.* |
| 34 | ♀ | 6 | 1½ drachms .. .. .. | .. | Darling .. .. | *Ibid.*, p. 1,340. |
| 35 | ♀ | 9 | Last dose .. .. .. .. | .. | Burton-Fanning .. | *Ibid.*, 1901, i, 1,202. |
| 36 | | .. | .... | .. | Kiwull .. .. .. | *Zentralbl. f. innere Med.*, 1902, xxiii, 1,233. |
| 37 | ♀ | $2\frac{9}{12}$ | 30–40 drops .. .. .. | D | Dwelle .. .. .. | *J. Am. M. Ass.*, 1903, xli, 1,540. |
| 38 | | $1\frac{4}{12}$ | 4 drops repeated in two hours | .. | Dillard .. .. .. | *Therap. Gaz.*, 1903, xix, 221. |
| 39 | ♂ | 4½ | ? | .. | Filbert .. .. .. | *Amer. Med.* (Phila.), 1904, vii, 254. |
| 40 | ♀ | 4½ | Five drops .. .. .. | .. | Oberdorfer .. .. | *Arch. Pediat.*, 1903, xx, 842. |
| 41 | | .. | .... | .. | Roth .. .. .. | *Méd. inf.* (Paris), 1905, 452. |
| 42 | | 4 | A drachm .. .. .. .. | .. | Tresling.. .. .. | *Nederl-Tydschr. v. Geneesk.*, 1906, i, 788. |
| 43 | | .. | .... | .. | Cijfen .. .. .. | *Ibid.*, p. 1,068. |
| 44 | ♂ | $\frac{7}{12}$ | Five drops .. .. .. | .. | Lobl .. .. .. | *Wien. klin. Wchnschr.*, 1907, xx, 564. |
| 45 | ♂ | 2 | Four drops repeated in one hour | .. | Lobl .. .. .. | *Ibid.* |
| 46 | ♂ | 4 | .... | .. | Lobl .. .. .. | *Ibid.* |
| 47 | | $1\frac{9}{12}$ | Last dose .. .. .. .. | .. | Benson .. .. .. | *Brit. M. J.*, 1907, ii, 204. |
| 48 | | .. | Last dose .. .. .. .. | .. | Langdon .. .. | *Ibid.*, p. 1,648. |

### REFERENCES.

[1] *München. med. Wchnschr.*, 1889, xxxvi, 787.
[2] *Med. Rec.*, 1890, xxxviii, 257.
[3] *Berl. klin. Wchnschr.*, 1890, xxvii, 508.
[4] Inaug. Dissert., Zurich, 1894.
[5] *Rev. mens. d. Mal. de l'Enf.*, 1896.
[6] Hutchison and Collier, *Index of Treatment*, 1908, p. 850.
[7] *Proc. Roy. Soc. Med.*, 1909, ii, Sect. Dis. of Childr., p. 77.

The risk of decomposition can be lessened by keeping the drug from the sunlight, dispensing it in darkened bottles, and replacing the stopper immediately.

With all these precautions, the fear of poisonous effects arising is reduced to a minimum; but there will, no doubt, still remain a certain number of cases in which unpleasant effects are caused, and it seems of great importance that all these should be recorded, in order that a correct estimate may be formed as to whether the undoubted virtues of the drug are or are not counterbalanced by its drawbacks.

In the table given it may be useful to bear in mind that there are about eight "drops" of bromoform in a minim, and that about six minims weigh one gramme.

---

